# Intratumoral heterogeneity and chemoresistance in nonseminomatous germ cell tumor of the testis

**DOI:** 10.18632/oncotarget.13380

**Published:** 2016-11-16

**Authors:** Mehmet Asim Bilen, Kenneth R. Hess, Matthew T. Campbell, Jennifer Wang, Russell R. Broaddus, Jose A. Karam, John F. Ward, Christopher G. Wood, Seungtaek L. Choi, Priya Rao, Miao Zhang, Aung Naing, Rosale General, Diana H. Cauley, Sue-Hwa Lin, Christopher J. Logothetis, Louis L. Pisters, Shi-Ming Tu

**Affiliations:** ^1^ Department of Hematology and Medical Oncology, Winship Cancer Institute of Emory University, Atlanta, GA, USA; ^2^ Department of Biostatistics the University of Texas MD Anderson Cancer Center, Houston, Texas, USA; ^3^ Department of Genitourinary Medical Oncology the University of Texas MD Anderson Cancer Center, Houston, Texas, USA; ^4^ Department of Pathology the University of Texas MD Anderson Cancer Center, Houston, Texas, USA; ^5^ Department of Urology the University of Texas MD Anderson Cancer Center, Houston, Texas, USA; ^6^ Department of Radiation Oncology the University of Texas MD Anderson Cancer Center, Houston, Texas, USA; ^7^ Department of Investigational Cancer Therapeutics the University of Texas MD Anderson Cancer Center, Houston, Texas, USA; ^8^ Department of Translational Molecular Pathology, The University of Texas MD Anderson Cancer Center, Houston, Texas, USA

**Keywords:** testicular cancer, intratumoral heterogeneity, chemoresistance, nonseminomatous germ cell tumor, next-generation sequencing

## Abstract

**Background:**

Nonseminomatous germ cell tumor of the testis (NSGCT) is largely curable. However, a small group of patients develop refractory disease. We investigated the hypothesis that intratumoral heterogeneity contributes to the emergence of chemoresistance and the development of refractory tumor subtypes.

**Results:**

Our institution's records for January 2000 through December 2010 included 275 patients whose primary tumor showed pure embryonal carcinoma (pure E); mixed embryonal carcinoma, yolk sac tumor, and teratoma (EYT); or mixed embryonal carcinoma, yolk sac tumor, seminoma, and teratoma (EYST). Patients with EYST had the highest cancer-specific mortality rate (*P* = .001). They tended to undergo somatic transformation (*P* = .0007). Two of 5 patients with clinical stage I EYST who had developed recurrence during active surveillance died of their disease.

**Materials and Methods:**

In this retrospective study, we evaluated consecutive patients who had been diagnosed with the three most common histological phenotypes of NSGCT. Chemoresistance was defined as the presence of teratoma, viable germ cell tumor, or somatic transformation in the residual tumor or the development of progressive or relapsed disease after chemotherapy. In a separate prospective study, we performed next-generation sequencing on tumor samples from 39 patients to identify any actionable genetic mutations.

**Conclusions:**

Our data suggest that patients with EYST in their primary tumor may harbor a potentially refractory NSGCT phenotype and are at increased risk of dying from disease. Despite intratumoral heterogeneity, improved patient selection and personalized care of distinct tumor subtypes may optimize the clinical outcome of patients with NSGCT.

## INTRODUCTION

Intratumoral heterogeneity is pervasive in cancer and poses an obstacle to precision medicine; thus, it is critical to understand this phenomenon. Nonseminomatous germ cell tumor (NSGCT) of the testis provides a unique opportunity to elucidate the nature and implications of intratumoral heterogeneity in solid tumors. The pathological sample from NSGCT is usually complete and abundant. Its histological makeup is well established and self-evident. Its molecular profile is simple compared with that of other solid tumors [1]. In addition, its clinical course is relatively easy to trace and annotate.

Importantly, NSGCT is one of the most curable solid tumors [2]. More than 90% of patients diagnosed with NSGCT are cured. In our recent analysis of 615 patients, despite widespread metastases and increased tumor burden, about two thirds of patients with clinical stage IIIC NSGCT were cured with conventional treatments such as chemotherapy and surgery [3]. However, 5–10% of the patients in our study died of their NSGCT. Further analysis of the characteristics of tumors that are refractory to standard treatments will provide invaluable clues about chemoresistance in NSGCT and perhaps other solid tumors.

In many respects, NSGCT is a prototype cancer for studying intratumoral heterogeneity [4]. The pathogenesis of NSGCT recapitulates the embryogenesis of germ cells [5–7]. A specific chromosome change, namely isochromosome 12p, is observed in 86% of germ cell tumors and all of their histological components [8]. The molecular profiles of its various histological components, primary and metastatic tumors, stromal and epithelial compartments, and teratomatous and somatically transformed constituents are highly concordant [9–12]. Despite a common clonal origin and a similar genetic profile, it is striking that chemosensitive embryonal carcinoma and chemoresistant teratoma co-exist in a mixed NSGCT; these components are usually treated by chemotherapy and surgery, respectively. These observations indicate that intratumoral heterogeneity is intrinsic in NSGCT and suggest that a specific subtype may be responsible for the 5–10% of patients who die of their disease. Such cases might be found and their significance magnified in patients with early-stage NSGCT (i.e., clinical stage I or II at the time of diagnosis) who would be expected to have been cured and yet, despite standard treatments, died of their disease.

Previously, we demonstrated that intratumoral heterogeneity is caused in part by differentiation of pluripotent progenitor cells [3]. Importantly, we identified distinct subtypes of NSGCT that take into account intratumoral heterogeneity. In the present study, we focused on the clinical characteristics of the three most prevalent histological phenotypes that made up 44% of the NSGCTs in our population: EYT (24%), comprised of embryonal carcinoma, yolk sac tumor, and teratoma; pure E (11%), containing only embryonal carcinoma; and EYST (9%), composed of embryonal carcinoma, yolk sac tumor, seminoma, and teratoma. We determined their chemosensitivity versus chemoresistance and examined which phenotypes were at risk of developing refractory disease after chemotherapy. We investigated whether identification of distinct NSGCT phenotypes might improve selection of patients with clinical stage I disease for active surveillance, adjuvant chemotherapy, or retroperitoneal lymph node dissection (RPLND).

## RESULTS

### Histological phenotype and refractory disease analysis

Among the 615 patients evaluated in this study, the most common histological makeup in the primary tumor comprised embryonal carcinoma, yolk sac tumor, and teratoma (EYT) (149 patients, or 24%). The second most common histological makeup in the primary tumor was pure embryonal carcinoma (pure E) (68 patients, or 11%). The third most common histological makeup comprised embryonal carcinoma, yolk sac tumor, seminoma, and teratoma (EYST) (58 patients, or 9%). The 275 patients in these three groups constituted the population of the current study.

**Figure 1:**
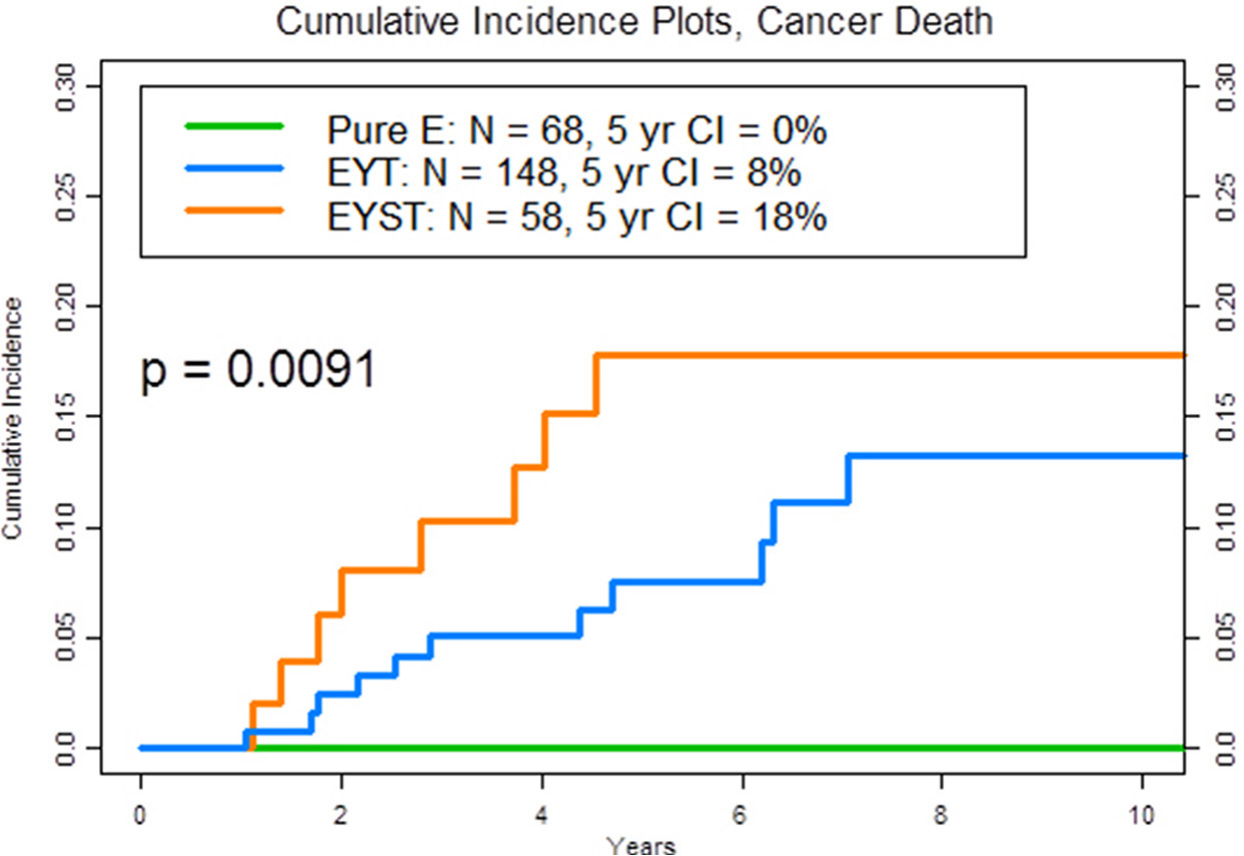
Plot of the 5-year cumulative incidence (CI) of cancer death by phenotype, according to the histological makeup of the primary tumor The three phenotypes are pure embryonal carcinoma (pure E) (green): embryonal carcinoma, yolk sac tumor, and teratoma (EYT) (blue): and embryonal carcinoma, yolk sac tumor, seminoma, and teratoma (EYST) (orange).

Table 1 lists the clinical characteristics of the 275 patients and the pathological properties of their 276 primary testicular tumors (1 patient had metachronous NSGCT). Notably, more patients with EYST had received salvage chemotherapy for progressive or relapsed disease (*P* = .0004), had had somatic transformation in their resected metastatic lesions (*P* = .0007), and had died from their EYST (18%) compared to patients with EYT (8%) and especially patients with pure E (0%) (*P* = .001) (Figure 1).

**Table 1:** Clinical characteristics and pathological properties for patients with nonseminomatous germ cell tumor (NSGCT) of the testis and distinct histological phenotypes

Characteristic	NSGCT Phenotype		
	Pure E	EYT	EYST
Total patients, n	68	149	58
Age, median years (range)	25 (16–57)	23 (12–47)	29 (19–53)
Race, n (%)			
White	52 (77)	101 (68)	39 (67)
Hispanic	15 (22)	40 (27)	18 (31)
African-American	0 (0)	6 (4)	1 (2)
Asian	1 (1)	2 (1)	0 (0)
Stage*, n (%)			
IA	7 (10)	52 (35)	19 (33)
IB	18 (26)	25 (17)	9 (16)
IS	2 (3)	12 (8)	4 (7)
IIA	15 (22)	12 (8)	5 (9)
IIB	5 (7)	13 (9)	6 (10)
IIC	2 (3)	5 (3)	2 (3)
IIIA	9 (13)	9 (6)	4 (7)
IIIB	7 (10)	9 (6)	4 (7)
IIIC	3 (14)	12 (8)	5 (9)
Size of primary tumor, median cm (range)	2.8 (0.65–12.0)	4.5 (0.5–17.8)	4.1 (2.0–20)
Therapy^, n (%)			
Salvage chemotherapy	0 (0)	14 (9)	12 (21)
-Progressive disease	0 (0)	6 (4)	8 (14)
-Relapse	0 (0)	8 (5)	4 (7)
High-dose chemotherapy with transplant support	0 (0)	3 (2)	2 (3)
Whole-brain radiation	0 (0)	3 (2)	0 (0)
RPLND	19 (28)	51 (34)	23 (40)
-Teratoma	7 (10)	36 (24)	9 (16)
-Somatic transformation	0 (0)	2 (1)	8 (14)
-Viable germ cell tumor	1 (1)	8 (5)	4 (7)
-No evidence of disease	11 (16)	5 (3)	2 (3)
Died, n (%)	1 (1)	15 (10)	11 (19)
Died of NSGCT	0 (0)	11 (7)	10 (17)
Unrelated cause	1 (1)	4 (3)	1 (2)
-MDS/AML	1 (1)	0 (0)	0 (0)
-Co-morbidities	0 (0)	2 (1)	0 (0)
-Trauma	0 (0)	0 (0)	1 (2)
-Unknown	0 (0)	2 (1)	0 (0)

E, embryonal carcinoma; EYT, embryonal carcinoma, yolk sac tumor, teratoma; EYST, embryonal carcinoma, yolk sac tumor, seminoma, teratoma; RPLND, retroperitoneal lymph node dissection; MDS/AML, myelodysplastic syndrome/acute myelogenous leukemia

*Time of diagnosis (orchiectomy).

^After standard chemotherapy.

Table 2 shows the recurrence rates of the 128 patients with clinical stage I (IA or IB) disease who chose active surveillance, received adjuvant chemotherapy, or underwent an RPLND. Overall, the recurrence rates for those patients with clinical stage I tumor who underwent surveillance or received adjuvant chemotherapy were 29% and 4%, respectively (*P*=.0005). Notably, among patients who developed recurrent disease on active surveillance, 2 of 5 (40%) patients with clinical stage I EYST and 4 of 20 (20%) patients with clinical stage I EYT or EYST died from their NSGCT.

**Table 2:** Recurrence rates of patients with clinical stage I NSGCT who chose active surveillance, received adjuvant chemotherapy, or underwent a retroperitoneal lymph node dissection (RPLND)

Outcome by stage and treatment strategy	NSGCT Phenotype			Total
	Pure E	EYT*	EYST	
Stage IA				
Surveillance	4	36	17	57
Recurrence (%)	2 (50%)	7 (19%)	2 (12%)	11 (19%)
Adjuvant chemo	2	14	2	18
Recurrence (%)	0	1 (7%)	0	1 (6%)
RPLND	1	0	0	1
Recurrence (%)	0	0	0	0
Stage IB				
Surveillance	6	10	5	21
Recurrence (%)	3 (50%)	6 (60%)	3 (60%)	12 (57%)
Adjuvant chemo	12	15	4	31
Recurrence (%)	0	1 (7%)	0	1 (3%)
RPLND	0	0	0	0
Recurrence (%)	0	0	0	0
Recurrence				
Surveillance	5/10 (50%)	13/46 (28%)	5/22 (23%)	23/78 (29%)
Adjuvant chemo	0/14 (0%)	2/29 (7%)	0/6 (0%)	2/49 (4%)
Total	25	75	28	128
Recurrence (%)	5 (20%)	15 (20%)	5 (18%)	25 (20%)
CSM (%)	0 (0%)	2 (13%)	2 (40%)	4 (16%)

E, embryonal carcinoma; EYT, embryonal carcinoma, yolk sac tumor, teratoma; EYST, embryonal carcinoma, yolk sac tumor, seminoma, teratoma; RPLND, retroperitoneal lymph node dissection; CSM, cancer-specific mortality in patients with recurrence

*Two patients without follow-ups.

Table 3 shows the clinical presentations and the pathological findings for those patients whose primary tumor showed pure E, EYT, EYST, and who underwent surgery to resect residual disease after chemotherapy. Chemosensitivity was identified by either a complete radiographic or pathological response. Evidence of drug resistance was demonstrated by the presence of teratoma, viable non-teratomatous germ cell element, or somatic transformation in the pathological specimens.

**Table 3:** Clinical and pathological findings for patients whose primary tumor showed pure embryonal carcinoma; embryonal carcinoma, yolk sac tumor, and teratoma (EYT); embryonal carcinoma, yolk sac tumor, seminoma, and teratoma (EYST), and who underwent surgery to resect residual disease after chemotherapy

		Chemosensitive			Chemoresistant		
Stage	*n*	RadiographicCR	Negative Pathology	Teratoma	Viable Germ Cell Tumor	Somatic Transformation	Cancer- Specific Death
Pure E							
IA	2	1	0	1	0	0	0
IB	3	1	1	1	0	0	0
IIA	15	14	1	0	0	0	0
IIB	5	3	1	1	0	0	0
IIC	2	0	1	0	1	0	0
IIIA	9	6	2	1	0	0	0
IIIB	7	2	3	2	0	0	0
IIIC	3	0	2	1	0	0	0
Total*	46	27	11	7	1	0	0
EYT							
IA	8	2	0	3	1	2	0
IB	7	2	0	3	1	1	2
IS	3	0	0	1	1	1	0
IIA^^^	10	3	0	7	0	0	0
IIB	13	3	2	6	2	0	1
IIC	5	0	0	3	1	1	2
IIIA	9	3	3	3	0	0	0
IIIB^#^	7	0	0	6	1	0	2
IIIC^+^	11	3	1	5	2	0	4
Total*	73	16	6	37	9	5	11
EYST							
IA	2	0	0	1	0	1	1
IB	3	0	0	0	1	2	1
IS	2	0	0	2	0	0	0
IIA	5	3	1	1	0	0	0
IIB^^^	3	1	0	2	0	0	1
IIC	2	0	0	1	1	0	1
IIIA	4	0	1	1	0	2	2
IIIB^#^	3	1	0	0	1	1	1
IIIC^+^	5	0	0	1	2	2	3
Total*	29	5	2	9	5	8	10

CR, complete response.

*Thirteen patients (2 with pure E, 9 with EYT, 2 with EYST) with clinical stage IS are excluded from the table because they had no radiographic disease before or after chemotherapy and did not undergo surgery.

^^^Four patients (2 with EYT, 2 with EYST) had RPLND followed by adjuvant chemotherapy, one patient with EYST never had post-chemotherapy RPLND, because of progressive disease.

^#^Two patients (1 with EYT, 1 with EYST) without follow-up; one patient with EYT never had post-chemotherapy surgery, because of progressive disease.

^+^Two patients (1 with EYT, 1 with EYST) never had post-chemotherapy RPLND, because of progressive disease; one patient with EYST had both viable germ cell tumor and somatic transformation.

Table 4 shows clinical characteristics of the 28 patients who developed progressive or relapsed NSGCT after chemotherapy. None had pure E in their primary tumor. A majority of the patients with EYT or EYST who underwent surgery to remove residual tumor after chemotherapy, had viable germ cell tumor or somatic transformation in the pathological specimens, and died of their NSGCT, including those patients who were initially diagnosed with a clinical stage I or II NSGCT.

**Table 4:** Clinical characteristics of patients who developed progressive or relapsed NSGCT after chemotherapy

Patients	Stage	Refractory disease	CSM (mo)	Pathology*
EYT				
1	IIIC	R	52	
2	IIIC	R	85	Viable GCT
3	IIIB	R	76	
4	IIB	R		Necrosis
5	IS	R		Viable GCT
6	IB	R	74	Transformation
7	IB	R	56	Viable GCT
8	IA	R		Viable GCT
9	IIIC	P	22	
10	IIIC	P	12	Viable GCT
11	IIIB	P	26	Transformation
12	IIC	P	35	Viable GCT
13	IIC	P	21	
14	IIB	P	28	Viable GCT
EYST				
15	IIIC	R	107	Teratoma
16	IIIB	R		Viable GCT
17	IIA	R		Transformation
18	IIC	R		Teratoma
19	IA	R	49	Transformation
20	IIIC	P	22	Viable GCT
21	IIIC	P	NA	
22	IIIC	P		Viable GCT
23	IIIB	P	24	Transformation
24	IIIA	P	17	Transformation
25	IIIA	P	34	Transformation
26	IIC	P	45	Viable GCT
27	IB	P	55	Transformation
28	IIB	P	13	

EYT, embryonal carcinoma, yolk sac tumor, teratoma; EYST, embryonal carcinoma, yolk sac tumor, seminoma, teratoma; P, progressive disease; R, relapsed disease; CSM, cancer-specific mortality; GCT, germ cell tumor; NA, not available.

*Pathology was not obtained for patients whose disease was fulminant and who did not undergo surgery because it was not clinically indicated.

### Exome sequencing

Thirty-nine patients whose primary tumors comprised pure E, EYT, or EYST were prospective enrolled in a separate laboratory study to examine whether the presence of genetic aberrations differed within the three NSGCT phenotypes. Next-generation sequencing was performed of the common coding regions (“hotspots”) of 50 genes in their primary and/or available metastatic tumors (Table 5). One patient with EYST had both his primary tumor and metastatic retroperitoneal lymph node tested. One patient with pure E had a *KIT* mutation involving exon 14. Another patient with EYST had a *KIT* mutation involving exon 17.

**Table 5:** Molecular profiles for 39 patients with NSGCT of the testis by histological phenotype

	NSGCT Phenotype		
	Pure E	EYT	EYST
Patients, n	12	19	8
Stage,* n			
IA	2 ^(2)^	4	2
IB	3	1	0
IS	0	2	0
IIA	3	2	0
IIB	2	5	2
IIIA	2	4 ^(3)^	1
IIIB	0	0	0
IIIC	0	1	3 ^(1)^
Tumor sample, n			
Testis	12 ^(2)^	15	4
RPLN	0	2 ^(3)^	2
LN, other	0	2	3 ^(1)^
Somatic mutations, n			
*KIT*, exon 17	0	0	1 ^(1)^
*KIT*, exon 14	1 ^(2)^	0	0
*PIK3CA*, exon 21	0	1 ^(3)^	0

E, embryonal carcinoma; EYT, embryonal carcinoma, yolk sac tumor, teratoma; EYST, embryonal carcinoma, yolk sac tumor, seminoma, teratoma; RPLN, retroperitoneal lymph node; LN, lymph node.

*Time of diagnosis (orchiectomy).

^(n)^Denotes specific patient. For example, patient 2 had stage 1A disease, and his primary (testis) tumor sample revealed a mutation in *KIT* exon 14.

Standardized nomenclature:

^(1)^NM_0002222.2(KIT):c.2447A > T p.D816V.

^(2)^14 NM_000222.2(KIT):c.2040T > G p.N680K.

^(3)^NM_006218.2(PIK3CA):c.3140A > G p.H1047R.

## DISCUSSION

Results of this study showed that patients whose primary tumor comprised the three most common NSGCT histological phenotypes (45% of our 11-year patient population) [3], i.e., pure E, EYT, or EYST, experienced disparate cancer-specific mortality rates at 5 years (Figure 1, P =.001). Although it is commonly assumed that recurrent clinical stage I NSGCT is very curable with standard chemotherapy and surgery (hence our rationale for active surveillance in all patients), certain patients with a particular tumor phenotype (i.e., EYST) and recurrent disease (i.e., clinical stage IB) may harbor potentially chemoresistant or refractory disease that becomes deadly when it is neglected or delayed and warrants an intensive surveillance or proactive treatment strategy (e.g., adjuvant chemotherapy).

Currently, active surveillance is advocated for patients with clinical stage I NSGCT on the basis of its safe approach, excellent cure rate, and overall low treatment burden [13]. However, a recurrence rate of about 30% was observed at 5 years after orchiectomy [13]. For patients whose primary tumor had showed lymphovascular invasion, presence of embryonal carcinoma, or rete testes invasion, the recurrence rate was 50%, and without any of these features, the recurrence rate was 12% [13]. Importantly, the 29% of patients who had developed recurrent disease received at least 3 courses of chemotherapy (e.g., BEP), and 8% underwent additional surgery apart from orchiectomy [13]. Identification of patients at increased risk of developing recurrent disease and especially of dying from it may enable prudent use of appropriate treatments.

We sought to determine whether intratumoral heterogeneity contributed to chemoresistance and whether a particular subtype of NSGCT benefited from a specific therapeutic strategy to maximize clinical outcome. Our results suggest that patients with clinical stage IA EYT or EYST might not benefit from adjuvant chemotherapy, because the recurrence rate of 6% on adjuvant chemotherapy was only slightly better than that of 17% for patients on active surveillance. In contrast, patients with clinical stage IB disease might benefit from adjuvant chemotherapy, because the recurrence rate of 5% for patients who received adjuvant chemotherapy was significantly better than that of 60% for patients on active surveillance (Table 2), *P* =.006.

Importantly, our data suggest that the risk of cancer-specific death from recurrent clinical stage I NSGCT might not be equal among the different phenotypes. Although the recurrence rate of patients with clinical stage I pure E was high (i.e., 50%), all of these patients were cured. However, 40% of patients with clinical stage I EYST and 20% of patients with clinical stage I EYT or EYST who developed recurrent disease on active surveillance died from their NSGCT. This observation has never been addressed in previous studies [13–15]. Paradoxically, increased recurrence rate did not translate to increased mortality rate in the pure E tumor phenotype. Therefore, we should be cognizant of a potential discordance between recurrence and mortality depending on the tumor subtype.

The results indicate that there may be ways to improve the selection of patients with clinical stage I NSGCT for active surveillance or adjuvant chemotherapy [16]. It is plausible that the different clinical outcomes are related to differential chemoresistance and to a greater propensity for certain distinct NSGCT subtypes to contain refractory phenotypes, such as presence of teratoma with viable non-teratomatous germ cell tumor or somatic transformation after chemotherapy. Our data suggest that certain patients with clinical stage I NSGCT on active surveillance who develop recurrence may have a propensity to harbor such chemoresistant tumors and are at increased risk of dying from disease. Additional studies are needed to validate this finding in a separate data base.

We found a preponderance of refractory disease in certain NSGCT phenotypes, i.e., EYT and EYST compared with pure E. Hence, 22 of 29 (76%) patients with EYST had evidence of drug resistance as demonstrated by the presence of teratoma with or without viable non-teratomatous germ cell element or somatic transformation in the residual pathological specimens after chemotherapy, whereas 7 of 29 (24%) patients had either a complete radiographic or pathological response (Table 3). In contrast, 8 of 46 (17%) patients with pure E had evidence of drug resistance, whereas 38 of 46 (83%) patients had either a complete radiographic or pathological response (Table 3), *P* < .0001. This observation is evident across all clinical stages, even though statistical significance could not be demonstrated for patients with clinical stage I disease due to the limited sample size and number of events.

Another way to assess chemoresistance in an otherwise extraordinarily chemosensitive and curable cancer such as NSGCT is to evaluate the nature of chemoresistance in the rare patients who developed refractory (i.e., progressive or relapsed) disease after chemotherapy (Table 4). This task was made possible and might be enhanced by focusing on a potentially lethal phenotype of NSGCT that contained yolk sac tumor in the primary tumor [3]. Importantly, refractory tumor and lethal NSGCT were not observed in any of our patients with pure E. However, viable germ cell tumor or somatic transformation was frequently detected in the residual tumor after chemotherapy in patients with refractory EYT (70% and 20%, respectively) or EYST (33% and 50%, respectively). Remarkably, 5 of 8 (63%) patients with refractory EYT and 4 of 6 (66%) patients with refractory EYST, who were initially diagnosed with a clinical stage I or II disease died of their NSGCT.

Previously, we did not detect any consistent gene mutation among potentially lethal NSGCT tumors in the entire coding region of 409 genes [3]. In the current prospective study, using next-generation exome sequencing of common coding regions (“hotspots”) of 50 genes, we found a somatic mutation in the same *KIT* gene but different exons of a patient who was cured with pure E and of another patient who died with advanced EYST (Table 5). It is plausible that unknown “driver” genetic defects could still be involved in the pathogenesis and potential lethality of a particular subtype of NSGCT [17, 18]. However, NSGCT is known to be relatively simple tumor with a markedly low rate of somatic mutations (21). In addition, certain malignant tumors do not have any putative driver mutations [19], while benign tumors and normal tissues do [20, 21]. Importantly, available data have demonstrated that such genetic aberrations are likely to be similarly distributed among the different components of a mixed NSGCT [9–12, 22, 23]. Further research is needed to elucidate the basic mechanism of chemoresistance in refractory NSGCT.

In summary, we demonstrated that distinct NSGCT phenotypes displayed different patterns of chemoresistance and disparate rates of cancer-specific mortality after chemotherapy. Certain patients with clinical stage I NSGCT who develop recurrence on active surveillance might be at increased risk of dying from disease. Despite intratumoral heterogeneity, improved patient selection and personalized care may maximize therapeutic efficacy in the management of different NSGCT phenotypes with curative intent.

## MATERIALS AND METHODS

### Histological phenotype and refractory disease analysis

The current analysis involves a subset of a previously published population of patients with NSGCT [3]. As described previously, using the Tumor Registry database at The University of Texas MD Anderson Cancer Center (Houston, Texas), we identified all consecutive cases of testicular cancer diagnosed from January 2000 to December 2010. Only patients with NSGCTs (i.e., mixed germ cell tumor, embryonal carcinoma, choriocarcinoma, teratoma, or yolk sac tumor) were included (patients with pure seminoma were excluded). Exclusion criteria were orchiectomy after chemotherapy, pathological sample not available for review, non-germ cell tumor (i.e., paratesticular tumor), age < 3 years with a pure yolk sac tumor or teratoma, and extragonadal germ cell tumor. For the 615 patients who met these criteria, we evaluated the specimens for histological makeup as described previously [3]. The present analysis, which has overlapping methods, evaluated the 275 patients found to have primary tumor phenotypes of EYT, pure E, or EYST.

After orchiectomy and staging, patients with clinical stage I disease either underwent RPLND, received adjuvant chemotherapy, or pursued active surveillance. Patients with stage II underwent RPLND or received adjuvant chemotherapy. Patients with stage III disease received adjuvant chemotherapy. We evaluated the pathological features of all specimens from postchemotherapy RPLND and metastasectomies. Specifically, we determined whether patients with certain histological makeups were found to have teratomas, contain viable germ cell tumors other than teratomas, or develop somatic transformation. Chemoresistance was defined as the presence of any of these elements in residual metastatic lesions after chemotherapy. Chemosensitivity was defined as the occurrence of no viable tumor or complete radiographic remission. The data from RPLND performed prior to chemotherapy for the purposes of diagnosis, staging, or therapy were analyzed separately. Clinical stage I tumors for which progression was identified more than 3 months after orchiectomy were defined as stage I disease with recurrence. Those that progressed within 3 months of orchiectomy were staged according to the highest stage of disease presentation before treatment. Salvage chemotherapy was defined as the use of any second-line chemotherapy, usually after first-line treatment regimens (i.e., BEP [bleomycin, etoposide, and cisplatin] or EP [etoposide and cisplatin]), for progressive or relapsed disease. Patients with progressive or relapsed disease were those whose disease required chemotherapy, not RPLND or metastasectomy, within or after 6 months of their last chemotherapy treatment, respectively. A change in chemotherapy regimen to consolidate a complete response was not considered salvage therapy. Adjuvant chemotherapy was also not counted as salvage therapy in the analysis.

Patients’ pathological reports, laboratory test results, and clinical histories were collected from MD Anderson's clinical data-management computer system. The dates of patients’ deaths were obtained from their medical records or the Social Security Death Index (http://ssdi.genealogy.rootsweb.com/). More than 99% of the pathological diagnoses were reviewed and confirmed by at least one pathologist who specialized in genitourinary malignancies at MD Anderson. If the pathological reports from both MD Anderson and an outside institution were available, the report from MD Anderson was used to maximize consistency. Both cancer-specific and overall mortality were assessed for this study. Patients with no evidence of active NSGCT who died as a result of other causes, such as treatment-related complications, accidents, or comorbidities, were included in the analysis. The survival duration was measured from the date of diagnosis to the date of death, or the most recent date of record if the patient was still alive. For the one patient with metachronous tumors, the survival duration was measured from the date of his first diagnosis of NSGCT.

### Exome sequencing

Between June 2014 and January 2016, 39 patients with a diagnosis of NSGCT were prospectively enrolled in a laboratory protocol (PA11–0852) for sequencing of the common coding regions (“hotspots”) of 50 genes in the tumor and available paired germline tissue (CMS-50 panel).

Pathologists in MD Anderson's Tissue Qualification Laboratory identified the optimal formalin-fixed, paraffin-embedded tissue blocks for the study. For each paraffin block, a hematoxylin and eosin (H&E)-stained slide and unstained sections were prepared. The tumor tissue was dissected from an unstained sequential section using the H&E slide as a template. DNA was then extracted from the dissected tumor using a QIAamp DNA FFPE Tissue Kit (Qiagen Inc) and used for sequencing of genes in a CMS-50 panel (Ion Proton System, Life Technologies). All procedures were well established for the testing of solid tumors [24].

### Statistical considerations

For the histological phenotype and refractory disease analysis, we estimated the cumulative incidence functions for NSGCT-related death, treating non-NSGCT-related death as a competing risk [25]. The differences between these functions were assessed using Gray's test for cause-specific death. Because of its bias for cancer-specific death due to competing risks from death without cancer, we did not use the Kaplan-Meier method in our calculations. The Fine-Gray proportional hazards regression analysis was used to assess the relationships between study factors and NSGCT-related death while treating non-NSGCT-related death as a competing risk [26]. Pearson's chi-square test and Fisher's exact test were used to compare proportions between independent samples. All statistical analyses were performed using TIBCO Spotfire S+ 8.0 software for Windows and StatXact-9 (Cytel Software Corporation).

### Regulatory issues

This study (PA14–0099 and PA14–0894) was approved by the institutional review board of MD Anderson.

## References

[1] Lawrence MS, Stojanov P, Polak P, Kryukov GV, Cibulskis K, Sivachenko A (2013). Mutational heterogeneity in cancer and the search for new cancer-associated genes. Nature.

[2] International Germ Cell (1997). Consensus Classification: a prognostic factor-based staging system for metastatic germ cell cancers. International Germ Cell Cancer Collaborative Group. J Clin Oncol.

[3] Tu SM, Bilen MA, Hess KR, Broaddus RR, Kopetz S, Wei C (2016). Intratumoral heterogeneity: Role of differentiation in a potentially lethal phenotype of testicular cancer. Cancer.

[4] E Rajpert-De Meyts, Kvist M, Skakkebaek NE (1996). Heterogeneity of expression of immunohistochemical tumour markers in testicular carcinoma in situ: pathogenetic relevance. Virchows Arch.

[5] Masters JR, Koberle B (2003). Curing metastatic cancer: lessons from testicular germ-cell tumours. Nat Rev Cancer.

[6] Tu SM, Lin SH, Logothetis CJ (2002). Stem-cell origin of metastasis and heterogeneity in solid tumours. Lancet Oncol.

[7] SM T (2010). Clinical perspectives and implications of a stem-cell theory of cancer, in Rosen ST (ed). Cancer Treatment and Research.

[8] Atkin NB, Baker MC (1982). Specific chromosome change, i(12p), in testicular tumours?. Lancet.

[9] Kernek KM, Ulbright TM, Zhang S, Billings SD, Cummings OW, Henley JD, Michael H, Brunelli M, Martignoni G, Foster RS, Eble JN, Cheng L (2003). Identical allelic losses in mature teratoma and other histologic components of malignant mixed germ cell tumors of the testis. Am J Pathol.

[10] Jones TD, Wang M, Sung MT, Zhang S, Ulbright TM, Eble JN, Beck SD, Foster RS, Anagnostou JJ, Conner C, Cheng L (2006). Clonal origin of metastatic testicular teratomas. Clin Cancer Res.

[11] Cheng L, Zhang S, Eble JN, Beck SD, Foster RS, Wang M, Ulbright TM (2012). Molecular genetic evidence supporting the neoplastic nature of fibrous stroma in testicular teratoma. Mod Pathol.

[12] Kum JB, Ulbright TM, Williamson SR, Wang M, Zhang S, Foster RS, Grignon DJ, Eble JN, Beck SD, Cheng L (2012). Molecular genetic evidence supporting the origin of somatic-type malignancy and teratoma from the same progenitor cell. Am J Surg Pathol.

[13] Daugaard G, Gundgaard MG, Mortensen MS, Agerbaek M, Holm NV, Rorth M, von der Maase H, Christensen IJ, Lauritsen J (2014). Surveillance for stage I nonseminoma testicular cancer: outcomes and long-term follow-up in a population-based cohort. J Clin Oncol.

[14] Kollmannsberger C, Tandstad T, Bedard PL, Cohn-Cedermark G, Chung PW, Jewett MA, Powles T, Warde PR, Daneshmand S, Protheroe A, Tyldesley S, Black PC, Chi K, So AI, Moore MJ, Nichols CR (2015). Patterns of relapse in patients with clinical stage I testicular cancer managed with active surveillance. J Clin Oncol.

[15] Gilbert DC, Al-Saadi R, Thway K, Chandler I, Berney D, Gabe R, Stenning SP, Sweet J, Huddart R, Shipley JM (2016). Defining a New Prognostic Index for Stage I Nonseminomatous Germ Cell Tumors Using CXCL12 Expression and Proportion of Embryonal Carcinoma. Clin Cancer Res.

[16] Pagliaro LC, Tannir NM, Tu SM, Logothetis CJ (2015). Management of Clinical Stage I Testicular Cancer: How Should We Define Success? J Clin Oncol.

[17] Collisson EA, Cho RJ, Gray JW (2012). What are we learning from the cancer genome?. Nat Rev Clin Oncol.

[18] Chen JC, Alvarez MJ, Talos F, Dhruv H, Rieckhof GE, Iyer A, Diefes KL, Aldape K, Berens M, Shen MM, Califano A (2014). Identification of causal genetic drivers of human disease through systems-level analysis of regulatory networks. Cell.

[19] Litchfield K, Levy M, Huddart RA, Shipley J, Turnbull C (2016). The genomic landscape of testicular germ cell tumours: from susceptibility to treatment. Nat Rev Urol.

[20] Mack SC, Witt H, Piro RM, Gu L, Zuyderduyn S, Stutz AM, Wang X, Gallo M, Garzia L, Zayne K, Zhang X, Ramaswamy V, Jager N (2014). Epigenomic alterations define lethal CIMP-positive ependymomas of infancy. Nature.

[21] Martincorena I, Roshan A, Gerstung M, Ellis P, P Van Loo, McLaren S, Wedge DC, Fullam A, Alexandrov LB, Tubio JM, Stebbings L, Menzies A, Widaa S (2015). Tumor evolution. High burden and pervasive positive selection of somatic mutations in normal human skin. Science.

[22] Burger H, Nooter K, Boersma AW, Kortland CJ, Stoter G (1997). Lack of correlation between cisplatin-induced apoptosis, p53 status and expression of Bcl-2 family proteins in testicular germ cell tumour cell lines. Int J Cancer.

[23] Cavallo F, Feldman DR, Barchi M (2013). Revisiting DNA damage repair, p53-mediated apoptosis and cisplatin sensitivity in germ cell tumors. Int J Dev Biol.

[24] Singh RR, Patel KP, Routbort MJ, Reddy NG, Barkoh BA, Handal B (2013). Clinical validation of a next-generation sequencing screen for mutational hotspots in 46 cancer-related genes. J Mol Diagn.

[25] RJ G (1988). A class of k-sample tests for comparing the cumulative incidence of a competing risk. Ann Stat.

[26] Fine JP GR (1999). A proportional hazards model for the subdistribution of a competing risk. J Am Stat Assoc.

